# Accelerated Production of Biopharmaceuticals via Microwave-Assisted Freeze-Drying (MFD)

**DOI:** 10.3390/pharmaceutics15051342

**Published:** 2023-04-27

**Authors:** Nicole Härdter, Raimund Geidobler, Ingo Presser, Gerhard Winter

**Affiliations:** 1 Department of Pharmacy, Pharmaceutical Technology and Biopharmaceutics, Ludwig-Maximilians-Universität München, 81377 Munich, Germany; nicole.haerdter@cup.uni-muenchen.de; 2 Boehringer Ingelheim Pharma GmbH & Co. KG, Pharmaceutical Development Biologicals, 88397 Biberach an der Riß, Germany

**Keywords:** microwave, freeze-drying, lyophilization, monoclonal antibody, excipients, stability

## Abstract

Recently, attention has been drawn to microwave-assisted freeze-drying (MFD), as it drastically reduces the typically long drying times of biopharmaceuticals in conventional freeze-drying (CFD). Nevertheless, previously described prototype machines lack important attributes such as in-chamber freezing and stoppering, not allowing for the performance of representative vial freeze-drying processes. In this study, we present a new technical MFD setup, designed with GMP processes in mind. It is based on a standard lyophilizer equipped with flat semiconductor microwave modules. The idea was to enable the retrofitting of standard freeze-dryers with a microwave option, which would reduce the hurdles of implementation. We aimed to collect process data with respect to the speed, settings, and controllability of the MFD processes. Moreover, we studied the performance of six monoclonal antibody (mAb) formulations in terms of quality after drying and stability after storage for 6 months. We found drying processes to be drastically shortened and well controllable and observed no signs of plasma discharge. The characterization of the lyophilizates revealed an elegant cake appearance and remarkably good stability in the mAb after MFD. Furthermore, overall storage stability was good, even when residual moisture was increased due to high concentrations of glass-forming excipients. A direct comparison of stability data following MFD and CFD demonstrated similar stability profiles. We conclude that the new machine design is highly advantageous, enabling the fast-drying of excipient-dominated, low-concentrated mAb formulations in compliance with modern manufacturing technology.

## 1. Introduction

To date, almost half of biopharmaceutical products are marketed in the form of dry, solid formulations [1], as they are not sufficiently stable in aqueous formulations over the intended shelf life [2,3]. Conventional freeze-drying (CFD), also known as lyophilization, is a well-established method of preserving sensitive protein drugs but comes with long process times and high energy consumption [3,4]. With emerging patient-centered drug manufacturing in the biopharmaceutical industry [5], small batch sizes create an increasing need for time-saving technologies and flexibility. Hence, numerous new drying technologies and approaches are being developed to speed up the lengthy process [1,6,7,8,9].

In this paper, we are neither focused on a particular formulation attempt to enable aggressive freeze drying [10,11,12], nor on the use of organic solvents [13,14,15] or continuous processes [16,17] but rather on a new solution to introduce sublimation energy faster and more effectively using microwaves. Due to the nature of microwave radiation, energy is directly transferred to the lyophilizates, resulting in volumetric heating [18]. This contrasts with CFD, where energy is delivered mainly through convection [19] and only partly through conduction and radiation. For more information on the basic principles of microwave-assisted freeze-drying (MFD), the reader is referred to [20]. While MFD is already widely used for quality foods [21,22], only a few studies have been published addressing its applications in bacterial cells [20,23]; vaccines and proteins [24,25]; and, specifically, monoclonal antibodies (mAbs) [26,27]. A few years back, Evans et al. first introduced the technology in the field of pharmaceuticals and demonstrated its applicability to mAbs and a model vaccine [28]. Following this, Gitter et al. evaluated the stability of two monoclonal IgG1-type antibodies and found comparable stability profiles following MFD and CFD [26,27]. More recently, Bhambhani et al. proposed a first-principle model investigating the principles of microwave-assisted freeze-drying of proteins and a vaccine [24]. Furthermore, a mechanistic model was proposed by Park et al. [29]. In [25], the statistical electromagnetics theory was used to create efficient and uniform heating for myoglobin samples. Nevertheless, these previously described machines come with several drawbacks: (1) Samples have to be frozen externally and subsequently transferred to the microwave dryer because shelf-freezing within the cabinet is not feasible. (2) In-chamber stoppering after lyophilization is not possible with prototypes, meaning that vials have to be stoppered by hand at atmospheric pressure. In [25], an auxiliary chamber was inserted into a lab-scale freeze-dryer, and it remains an open question whether the vials are in direct shelf contact during freezing and if machine-stoppering the vials inside this chamber is possible. However, these features are indispensable for implementation in a GMP environment. There is a definite need for much better-controlled processes (i.e., the loading of the vials using proven systems or freezing and stoppering within the chamber) to avoid external side effects such as particulate entry, temperature variations, water absorption until container closure, and associated increased residual moisture contents.

This work addresses the challenges identified and presents a new setup for microwave-assisted freeze-drying (MFD), which combines the advantages of a regular GMP lyophilizer with flat and scalable microwave radiation sources. The results of our investigation display the new setup and its performance in two aspects: (A) Process data were collected with respect to microwave settings, drying speed, and controllability. (B) The quality of the dried products after drying and their stability after storage were assessed. Accordingly, we examined six different formulations of a monoclonal antibody (mAb) in the new drying setup. The antibody was formulated in a generic and low concentration in the presence of a typical histidine buffer and a commonly used surfactant (i.e., polysorbate 20). To study the effect of the solid content, we added different sugar types, namely, sucrose and trehalose, in two concentrations. Moreover, we investigated the dryability of non-standard matrices in MFD using 2-hydroxypropyl-beta-cyclodextrin (HP-β-CD) and arginine phosphate. Finally, we compared the stability profiles of the samples following the two drying protocols, i.e., MFD and CFD. The solid-state properties of the lyophilizates, as well as the physical and chemical stability of the mAb, were investigated at 4 °C, 25 °C, and 40 °C over the course of 6 months.

The new microwave-assisted freeze-drying (MFD) setup meets the requirements of modern manufacturing technology and is based on a typical laboratory-scale freeze-dryer with stainless steel shelves allowing for temperature control via silicon oil circulation. Chamber geometry, condenser, and cooling and vacuum systems represent the regular state of the art. Therefore, freezing, drying with and without microwave radiation, and stoppering under a partial vacuum can be conducted easily. Furthermore, neither the microwave source nor the product is rotated in this MFD setup: a phase shift is applied repeatedly to avoid the formation of cold and hot spots. With this work, we aim to provide a proof of concept for this new technology setup, but further process optimization is beyond the scope of the study.

## 2. Materials and Methods

### 2.1. Monoclonal Antibody and Chemicals

A monoclonal IgG type 1 antibody (mAb) was used in the study. L-histidine monohydrochloride monohydrate (99% purity) and L-histidine (cell culture reagent) were purchased from Alfa Aesar (Ward Hill, MA, USA). D(+)-trehalose dihydrate (97.0–102.0% purity) Ph. Eur., NF certified, was purchased from VWR International (Radnor, PA, USA). EMPROVE^®^ exp sucrose, EMPROVE^®^ exp di-sodium hydrogen phosphate dihydrate, EMPROVE^®^ bio sodium chloride, and EMSURE^®^ ortho-phosphoric acid (85%) were purchased from Merck KGaA (Darmstadt, Germany). Trizma^®^ base and Trizma^®^ hydrochloride (both in BioXtra grade), (2-Hydroxypropyl)-β-cyclodextrin produced by Wacker Chemie AG, L-arginine BioUltra (≥99.5%), and sodium azide (≥99.5%) were purchased from Sigma Aldrich (Burlington, MA, USA). Sodium dihydrogen phosphate dihydrate (99%) was purchased from Grüssing GmbH (Filsum, Germany). Super Refined™ Polysorbate 20-LQ-(MH) was purchased from Croda (Edison, NJ, USA). For the preparation of all solutions, ultrapure water from an Arium^®^ system from Sartorius Lab Instruments GmbH (Goettingen, Germany) was used.

### 2.2. Preparation of the Formulations

The first experiments were carried out with 8% (*w*/*v*) and 10% (*w*/*v*) sucrose placebo formulations. Next, we continued with six verum formulations (Table 1). The bulk solution of the mAb was dialyzed and concentrated using a Minimate™ Tangential Flow Filtration (TFF) capsule (MWCO 30 kDa; Pall Corporation, New York, NY, USA). After extensive dialysis using a 7-fold excess of 10 mM of histidine buffer (pH 5.5), the final buffer consisted of 10 mM of histidine and 0.04% (*w*/*v*) polysorbate 20. The concentration of the mAb was determined with a Nanodrop 2000 UV spectrophotometer (Thermo Fisher Scientific, Waltham, MA, USA) at 280 nm, using the molar extinction coefficient. Stock solutions of the excipients were prepared with 10 mM of histidine buffer and mixed with the protein solution according to the intended composition (Table 1). Then, all formulations were sterile-filtered using 0.22 µm Sartolab^®^ RF polyethersulfone vacuum filtration units (Sartorius AG, Goettingen, Germany). For each formulation, 63 10R FIOLAX vials (MGlas AG, Muennerstadt, Germany) were filled with 5 mL of the respective solutions and semi-stoppered with lyophilization stoppers (Flurotec^®^ laminated rubber stoppers, West Pharmaceutical Services, Inc., Exton, PA, USA).

### 2.3. Freeze-Drying Process

A laboratory-scale freeze-dryer by OPTIMA Pharma GmbH (Schwäbisch Hall, Germany) equipped with flat, emitting semiconductor microwave modules was used for all lyophilization runs. Due to the experimental nature of the new technical setup, the experiments had to be carried out in the technical workshop of the machine manufacturer. The vials were arranged in a hexagonal array (180 mm × 190 mm) in the middle of a shelf (486 mm × 440 mm) of the freeze-dryer. The microwave modules were mounted below the shelf above the vials (antenna area approximately 26 cm × 26 cm) and showed high mechanical stability to enable the stoppering of the vials after drying. Freezing to a final shelf temperature of −50 °C was carried out as suggested by Tang et al. [4]. Primary drying was conducted at a shelf temperature of −15 °C and then increased to 30 °C for secondary drying and held for 6 h (chamber pressure, 50 μbar; all ramps, 1 K/min). For MFD, 2 × 90 W (2.43–2.48 GHz) was applied during drying. For this purpose, microwave radiation was started as soon as the intended vacuum for primary drying was established (to decrease the risk of local plasma emergence [30]) and ran continuously until the shelf temperature reached 0 °C to not overheat the samples. The most commonly used temperature sensors, i.e., thermocouples and resistance temperature detectors [31], malfunction in electromagnetic environments. For this reason, fiber-optic temperature sensors (Weidmann Technologies Deutschland GmbH, Dresden, Germany) were employed for product temperature recording for both MFD and CFD. To monitor the drying process, a mass spectrometer (Pfeiffer Vacuum GmbH, Asslar, Germany) was used in addition to comparative pressure measurement via a Pirani and MKS Baratron gauge. After the completion of the drying process, vials were stoppered under a partial vacuum in a nitrogen atmosphere and crimped with Flip-Off^®^ seals (West Pharmaceutical Services, Inc., Exton, PA, USA).

### 2.4. Karl–Fischer Titration

Coulometric Karl–Fischer titration was used to determine the residual moisture content (rM) of the lyophilizates of F1–F6. Under controlled humidity conditions (relative humidity < 10%), the cakes were gently crushed, and 40–70 mg of each cake was transferred into 2R vials. Afterward, the samples were placed in an oven (temperature 100 °C), and the extracted water was transferred to the coulometric titration cell with a dry carrier gas flow (Aqua 40.00 Vario Plus, ECH Elektrochemie Halle GmbH, Halle (Saale), Germany). Relative moisture content was calculated (%, *w*/*w*). Prior to sample analysis, equipment performance was verified by measuring the Apura^®^ water standard oven 1% (Merck KGaA, Darmstadt, Germany) in triplicate.

### 2.5. Frequency Modulated Spectroscopy (FMS)

A Lighthouse FMS-1400T (Lighthouse Instruments, Charlottesville, VA, USA) was used to perform a 100% headspace moisture analysis after lyophilization. Samples were kept refrigerated and subsequently equilibrated at room temperature for at least 3 h before analysis. Headspace moisture data are provided as partial pressures in mbar. Nitrogen was used as a buffer gas to remove background noise due to ambient air moisture, and samples were equilibrated in the device for 15 s before the measurements were started. Before sample analysis, a system suitability test was conducted using five standards covering an appropriate moisture range.

### 2.6. Brunauer–Emmet–Teller (BET) Krypton Gas Adsorption

The specific surface area was determined according to Brunauer–Emmet–Teller (BET) using krypton gas adsorption in a liquid nitrogen bath at 77 K (Autosorb 1, Quantachrome, Boynton Beach, FL, USA). At least 100 mg of the gently crushed samples was used to fill the 9 mm sample cells under controlled humidity conditions (relative humidity < 10%). An outgassing step was performed for at least 2 h at room temperature prior to analysis. Gas adsorption was determined for 11 measuring points, covering a relative pressure ratio of 0.05–0.30. The specific surface area was determined using the multipoint BET method fit in the Autosorb 1.55 software.

### 2.7. Scanning Electron Microscopy (SEM)

The morphology of the freeze-dried cakes was analyzed via scanning electron microscopy (SEM) using a Helios NanoLab G3 UC (FEI, Hillsboro, OR, USA) at an acceleration voltage of 2 kV. Fragments of the top and bottom layers of the lyophilizates were extracted in a glove box (relative humidity < 10%). Subsequently, the samples were sputtered with carbon (10 nm layer thickness) using a CCU-010 HV sputterer (Safematic GmbH, Zizers, Switzerland). Images were taken at 175-fold magnification.

### 2.8. Micro-Computed Tomography (µ-CT)

Noninvasive 3-dimensional micro-computed tomography (µCT) using a Skyscan 1273 X-ray microtomograph (Bruker, Billerica, MA, USA) was used to obtain global information on the cake structure. The lyophilizates were measured without further processing at an acceleration voltage of 70 kV and a beam current of 114 µA. The image pixel size is 6.5 µm/voxel. To reduce beam hardening effects related to the vial geometry, a flat field acquisition in the headspace of the vials was carried out prior to each measurement. An exposure time of 345 ms with 6 averages per projection was applied. The samples were rotated over 360° with a step size of 0.15°. Image reconstruction and analysis were carried out using the NRecon 1.7.5.1 and CTAnalyzer 1.20.8.0 software, respectively.

### 2.9. X-ray Powder Diffractometry (XRPD)

An ARL EQUINOX X-ray diffractometer (Thermo Fisher Scientific, Waltham, MA, USA) was used to determine the solid state of the lyophilized samples. The device operated with Cu-Kα_1_ and Cu-Kα_2_ radiation (λ = 0.15417 nm) at 40 kV and 0.5 mA. Detection was carried out with a curved counting wire detector with flowing counting gas (angular range, 110° 2-θ); the radiation source was a microfocus X-ray tube with mirror optics. Prior to analysis, the lyophilized cakes were gently ground into powder and placed on brass sample holders. Adhesive tape was used to seal the sample holders immediately after sample mounting to protect the moisture-sensitive powders from the surrounding air. Powder diffraction scans were conducted in a 2-θ range of 5° to 45° (0.03° steps).

### 2.10. Reconstitution of the Lyophilizates

Reconstitution of the lyophilizates was performed by adding ultrapure water. The required volume was calculated individually for all formulations to match the volume of water removed during freeze-drying.

### 2.11. Size Exclusion Chromatography (SEC)

For the quantification of monomer yield and protein aggregates, we used a Thermo Scientific™ Dionex™ UltiMate™ 3000 UHPLC system equipped with a VWD-3400RS UV/Vis absorbance detector, all from Thermo Fisher Scientific (Waltham, MA, USA), and a TSKgel G3000SWxl, 7.8 × 300 mm, 5 µm column (Tosoh Bioscience, Tokyo, Japan). The running buffer was composed of 100 mM of sodium phosphate, 300 mM of sodium chloride, and 0.05% (*w*/*v*) sodium azide, pH 7.0. Separation was performed at a flow rate of 1 mL/min, and 10 µL was injected. The elution of the reconstituted lyophilizates was detected by absorption at 280 nm, and, subsequently, the chromatograms were integrated using Chromeleon™ 7.2.7 (Thermo Fisher Scientific, Waltham, MA, USA). The relative monomer yield was calculated in relation to the amount of monomer prior to freeze-drying the respective formulations. The relative amount of high-molecular-weight species (HMWS) was calculated according to Svilenov et al. [32].

### 2.12. Cation Exchange Chromatography (CEX)

The chemical stability of the mAb was analyzed using a Thermo Scientific™ Dionex™ UltiMate™ 3000 UHPLC system equipped with a VWD-3400RS UV/Vis absorbance detector and a ProPac™ WCX-10G BioLC™ analytical column (4 × 250 mm) equipped with a ProPac™ WCX-10G BioLC™ guard column (4 × 50 mm), all from Thermo Fisher Scientific (Waltham, MA, USA). Mobile phase A consisted of 20 mM TRIS (pH 8.0), whereas mobile phase B contained 20 mM of TRIS and 300 mM of sodium chloride (pH 8.0). Elution was performed in a linear salt gradient mode from 0% B to 20% B in 30 min with a flow rate of 1 mL/min. Before analysis, the samples were diluted to a mAb concentration of 0.1 g/L with mobile phase A, and the injection volume was 100 µL. Elution was detected at 280 nm, and the integration of the chromatograms was performed with Chromeleon™ 7.2.7 (Thermo Fisher Scientific, Waltham, MA, USA). The peak areas were divided into three components: the main peak, acidic variants corresponding to every peak eluting before the main peak, and basic variants corresponding to every peak eluting after the main peak.

### 2.13. Flow Imaging Microscopy

The formation of subvisible particles was analyzed with a FlowCam 8100 (Fluid Imaging Technologies, Inc., Scarborough, ME, USA). The device was equipped with a 10 × magnification flow cell (80 µm × 700 µm) and operated using the VisualSpreadsheet^®^ 4.7.6 software. A 150 µL sample was analyzed at a flow rate of 0.15 mL/min, and particle images were taken at an auto image frame rate of 28 frames/s. Settings specified for particle identifications were at a 3 µm distance to the nearest neighbor and particle thresholds of 13 and 10 for dark and light pixels, respectively. The size of the particles was reported as the equivalent spherical diameter.

## 3. Results and Discussion

### 3.1. Effects of Microwave Assistance on the Freeze-Drying Process

**Figure 1:**
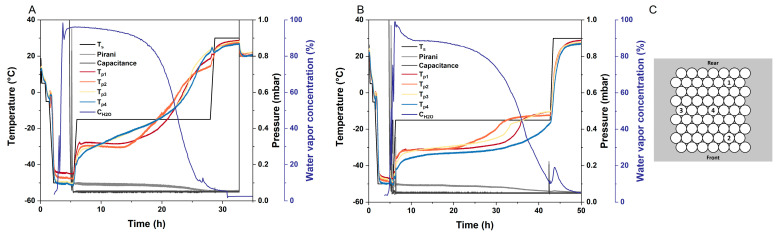
Readouts of the drying processes of a 10% (m/V) sucrose formulation. T_s_ represents the shelf temperature; the chamber pressure is monitored via a Pirani gauge (Pirani) and an MKS Baratron gauge (Capacitance); T_p_ is the readout of the fiber optic temperature sensors; and the water vapor concentration (C_H2O_) during drying is recorded with a mass spectrometer. (**A**) Microwave-assisted freeze-drying was conducted at 2.43 GHz–2.48 GHz and 180 W. (**B**) Conventional freeze-drying was performed using the same lyophilizer. (**C**) Position of the four fiber optic temperature probes and arrangement of the vial package on the shelf.

The first and most obvious effect of MFD is its potential to drastically reduce drying times. With microwave assistance, a 10% (m/V) sucrose formulation was dried within approximately 27 h, while it took about 44 h to dry it in a conventional manner. For both MFD and CFD, the vials were arranged in a similar setup on the shelf (Figure 1).

A second interesting effect is the distribution of heat in MFD. When comparing the temperature profiles of MFD and CFD, a drastic difference can be found: the typically observed edge effect is reversed in MFD, and center vials temporarily run equal to or warmer than edge vials (Figure 1A,B). After MFD, the residual moisture of the center vials was significantly lower (headspace moisture, 1.72 mbar ± 0.29 mbar) than in the edge vials (headspace moisture, 2.23 mbar ± 0.29 mbar), *p* < 0.05 (Figure S1). For more information on non-destructive headspace moisture analysis, allowing for other subsequent analytics on the very same sample vial, the reader is referred to [33]. Furthermore, a distinct spread between the product temperature readings toward the end of primary drying, as observed for CFD, is not found in MFD. Likewise, we observed slightly lower residual moisture levels in the center vials (1.41% ± 0.12%) compared with the edge vials (1.57% ± 0.13%) following CFD due to cooling radiation effects from the chamber walls, when the shelf temperature is increased during secondary drying (*n* = 7, [34]). The edge vial effect caused by differences in heat transfer during primary drying in CFD is an important issue that needs to be taken into account during cycle development as well as in scale-up, and this often leads to long, conservative drying cycles [35,36]. As our data indicated, this limitation can be overcome due to better energy distribution in MFD. It is in accordance with findings from Bhambhani et al., who found no constraint in energy input due to vial heat transfer, K_v_, when MFD is used [24]. The equalization of heat transfer in the center and corner vials is a promising effect and needs to be evaluated further to identify optimal frequencies and phase settings.

Thirdly, another peculiarity of MFD can be observed when looking at the process phases. When the shelf temperature is increased for secondary drying, the increased pressure reading from the Pirani gauge and water vapor concentration detected by mass spectrometry indicate a fair amount of residual water in the vials at that point in CFD (Figure 1B). These high levels cannot be found in MFD (Figure 1A). We, therefore, assume that separated primary and secondary drying does not exist in MFD. Moreover, the dielectric properties of frozen and liquid water are very different [37,38,39]. While ice shows a low dielectric loss factor [20], microwaves probably excite the highly polarizable unfrozen water [29]. This allows for faster and more robust drying processes since the glass transition temperature of the freeze concentrate thereby increases as the drying process progresses. The strength of MFD technology is often described as having the potential to increase heat transfer via volumetric heating and, therefore, overcome the bottleneck of CFD in heat transfer [20,24]. Apart from that, efforts toward robust formulations enabling fast and aggressive CFD have been made [10,12,40,41]. However, the aggressive drying of low-concentration protein formulations lacking crystalline bulking agents results in a poor macroscopic appearance [12,40,42]. Therefore, we particularly see the strength of MFD technology, among other things, in the fact that the processing of “difficult-to-dry” formulations, i.e., low T_g_’ and T_c_, combined with high filling volumes, can be conducted very fast as a result of increasing T_g_’ during drying.

### 3.2. Effects of the Excipients and Solute Concentration

To better understand how MFD processes work, we next aimed to examine the effect of solute concentration using two sugars, namely, sucrose and trehalose (Table 1, F1–F4), which are two of the most prominent stabilizers in the field of lyophilization [43]. While F1 and F3 with 8% (m/V) sugar represent commonly used concentrations for protein stabilization, 16% (m/V) sugar containing the formulations F2 and F4 are considered to be particularly difficult to dry using CFD. The reason for this is that high solute concentrations lead to increased mass transfer resistance, especially when combined with high filling volumes, which constitute a worst-case scenario, resulting in long drying times in CFD. Here, we observed that this relationship is different in MFD: despite the fact that higher dry-layer resistances in the cakes must be overcome, an increased solute content enhances dielectric heating. With MFD, F1 was dried within 28.5 h, while it took 55.7 h without microwaves (CFD) when the same protocol was applied. An increase in sugar concentration by factor two resulted in even shorter microwave-assisted drying times (26.4 h for F2) due to the lower amount of water that needed to be removed. The same is true for trehalose-based formulations F3 and F4 (29.1 h and 26.9 h drying time with MFD, respectively). The ability to enhance microwave absorption efficiency due to a higher solute concentration is in accordance with recently published work [24]. Furthermore, the amount of unfrozen water depends strongly on the composition of the formulation and correlates with the concentration of amorphous solutes [44,45]. Accordingly, MFD efficacy further increases with an increasing quantity of highly polarizable unfrozen water being excitable in F2 and F4 compared with F1 and F3.

To further study the effects of excipients, we investigated stabilizers that are less frequently used than disaccharides in the new MFD setup. More recently, the addition of cyclic oligosaccharide 2-hydroxypropyl-beta-cyclodextrin (HP-β-CD) was shown to provide stable formulations of monoclonal antibodies following aggressive CFD protocols [10,11]. Due to the high T_g_’ and T_c_ of such formulations [46,47], elegant cakes were obtained while HP-β-CD remained fully amorphous [48]. The first results from our group in a different MFD setup also indicated the applicability of HP-β-CD for MFD [26]. In this study, a binary mixture of HP-β-CD and sucrose was used (Table 1, F5), as proposed by Haeuser et al. [10]. The drying time with MFD was 29.9 h, and we believe that this can be even further reduced by applying higher shelf temperatures.

Since dipole rotation/vibration is a major mechanism in most biological materials resulting in heating due to microwave radiation [20], we were interested in studying an arginine-based formulation (Table 1, F6). Due to its pK_a_ of 13.8 [49], arginine is positively charged in acidic, neutral, and most basic formulation conditions [50]. The drying time of arginine-based formulation F6 (Table 1) was 31.5 h and thus did not differ much from the disaccharide-based formulations.

### 3.3. Solid State Properties of the Lyophilizates

**Figure 2:**
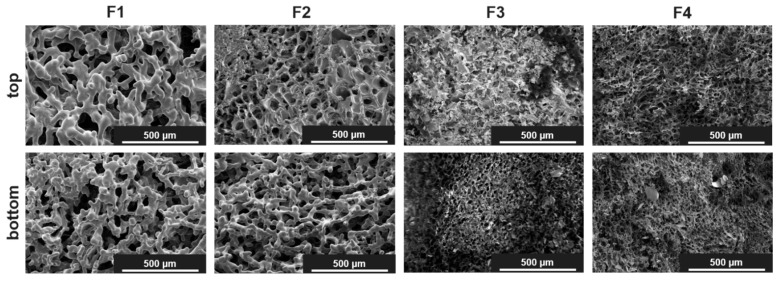
Representative SEM pictures from the top and bottom of the cakes after MFD at 175-fold magnification.

**Figure 3:**
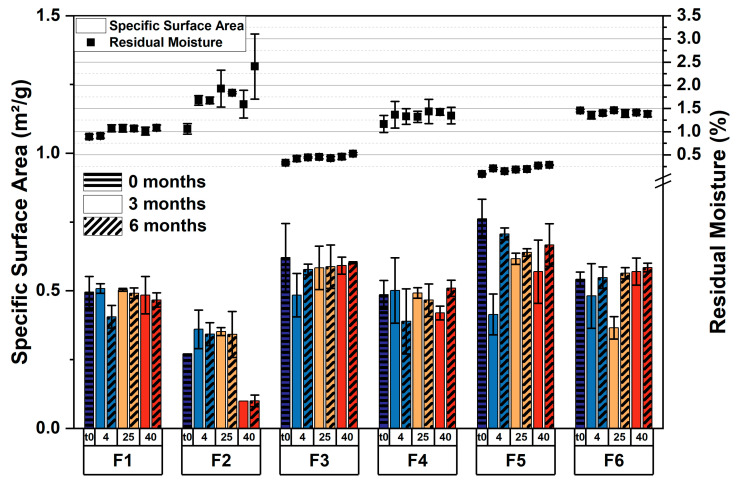
Solid-state properties of lyophilized formulations. Specific surface area (bars) and the respective residual moisture data (symbols) were obtained directly after lyophilization and during the stability study over the course of 6 months. The values are means (*n* = 2 for SSA; *n* = 3 for rM) ± standard deviation. Storage temperatures: 4, 25, and 40 °C.

The obtained cakes looked elegant on a macroscopic scale. Only for F2 was shrinkage in the cakes observed. On a microscopic scale, a cellular pore structure was found with scanning electron microscopy (SEM) for F3–F6, whereas F1 and F2 appeared to be microcollapsed (Figure 2 and Figure S2). However, due to the low T_g_’ values of the sucrose formulations, microcollapse may not be avoided with fast and aggressive drying [11]. This phenomenon is not related to the application of microwaves, and we likewise observed a microcollapsed structure for F1 after CFD [34]. Apart from that, the top and bottom showed a similar structure in SEM, even when sugar-rich formulations were dried. These findings align well with a micro-computed tomography (µCT) analysis of F1–F4, which revealed very similar pore size distribution for the respective pairs, i.e., 8% and 16% sugar (Figure S3). For the sucrose containing formulations F1 and F2, pore size was found to be shifted toward larger pores due to the aforementioned microcollapse. Furthermore, the specific surface area (SSA) indicated that the porous cake structures were retained throughout the stability study, except for F2 after 6 months at 40 °C, where further shrinkage appeared (Figure 3, bars). This is in good agreement with the residual moisture, which was low in all formulations after MFD (Figure 3, symbols) and remained constant over the course of 6 months except for fluctuations in the sucrose-rich formulation (F2). We, therefore, assume that, with F2, even the MFD technology is stretched to its limits, whereas the trehalose-rich formulation F4 showed no limitations. Furthermore, we used X-ray powder diffractometry (XRPD) to study the structural patterns of the lyophilizates. All formulations were fully amorphous after MFD (Figure S4) and storage at 40 °C for 6 months (exc. F2; [51]).

### 3.4. Storage Stability of the Formulations

Physical and chemical protein stability was determined after MFD and after storage at 4 °C, 25 °C, and 40 °C over the course of 6 months, respectively. It is important to emphasize that all formulations demonstrated remarkably good stability directly after MFD, which is not self-evident with regard to the aggressive drying conditions. The relative monomer yield remained constant within 6 months of storage for all formulations (Table 1 and Tables S1–S3), except for F2, which was stored at 40 °C (75.5% ± 0.2% after 6 months). This can be explained by the low glass transition temperature (T_g_) of the sugar-rich formulation (51.4 °C ± 2.1 °C). The excipients also had an impact on the formation of high-molecular-weight species (HMWS), i.e., soluble protein aggregates, during storage. The relative number of soluble aggregates was low for all formulations stored at 4 °C and 25 °C (Table 1, Tables S1 and S2). With 21.07% ± 0.08%, the highest number of aggregates was detected for F2 after storage for 6 months at 40 °C, while the mAb was preserved very well in F1, F3, and F4 at this temperature (Table 1 and Table S3). A slight increase in HMWS was observed for F5 (1.09% ± 0.02%) and F6 (3.34% ± 0.06%) after 6 months at 40 °C (Table 1).

The formation of larger, insoluble aggregates (≥25 µm and ≥10 µm) was low for F1–F5 after MFD and storage (Figure S5). In F6, the formation of aggregates was induced during drying, resulting in higher particle counts right from the start of the stability study (Figure S5). Although the ability of arginine salts to prevent protein aggregation has been published before and was confirmed in recent studies [50,52], we observed that, in the presence of microwave irradiation, its protective effect seems to be diminished, probably due to strong ion–dipole interactions between the microwave field and arginine salts, resulting in increased local heating. Further studies are needed to investigate whether this effect is also observed in other proteins and what effect other charged molecules or amino acids in MFD have. The number of subvisible particles (≥1 µm) was found to be at a relatively high level throughout the study but within the range of placebo formulations.

The chemical stability of the mAb was assessed with weak cation exchange (CEX) chromatography (Table 2). Directly after MFD, the relative number of acidic, i.e., deamidated, and basic species was found to be within the standard deviation of the liquid bulk [51]. Although a microcollapsed structure was observed for F1 and F2, only slight chemical changes were detected after storage for 6 months at 4 °C and 25 °C, respectively. After 6 months at 40 °C, acidic variants did not change significantly, and basic variants increased slightly from 13.7% ± 0.3% after lyophilization to 15.5% ± 0.3% for F1. For F2, acidic variants increased from 25.8% ± 0.1% to 30.5% ± 1.6%, and basic variants changed from 13.1% ± 0.3% to 20.9% ± 0.7% at 40 °C storage temperature. For the trehalose-based formulations, F3 and F4, the relative number of acidic species differs little from the amount directly after MFD, even when stored at 40 °C (F3, 26.0% ± 0.1% after MFD and 28.3% ± 1.0% after 6 months; F4, 24.3% ± 0.6% and 28.1% ± 2.4%), indicating very robust chemical stability over time. We observed somewhat more pronounced changes in the basic species of F3 (14.5% ± 0.3% after MFD, 20.5% ± 0.6% after storage at 40 °C) and F4 (11.0% ± 0.6% after MFD, 18.2% ± 1.4% after storage at 40 °C). However, chemical changes are in the same range for F3 and F4, suggesting that the mAb is equally well stabilized, and high trehalose concentrations do not limit MFD. Formulations F5 and F6 appeared less stabilizing at higher storage temperatures, i.e., at 25 °C and 40 °C. The relative number of acidic variants of F5 increased from 27.8% ± 0.1% to 32.9% ± 0.8% at 40 °C, and basic variants increased from 11.6% ± 0.6% to 25.7% ± 1.8%. The arginine phosphate that contained formulation F6 showed a noticeable increase in both acidic (27.2% ± 1.0% after MFD and 43.7% ± 0.2% after storage at 40 °C) and basic species (11.8% ± 1.9% and 21.4% ± 0.3%).

Since sugar-rich formulations are typically difficult to dry in CFD, we directed particular attention to the storage stability of these formulations following MFD and compared it with lower amounts of the same disaccharide. We, therefore, deliberately refrained from simultaneously increasing the mAb concentration, as the robustness of the lyophilization process correlates with the protein concentration [40]. Accordingly, we tested the most prominent used disaccharides, sucrose and trehalose, in two concentrations. The results of this study showed good storage stability for F1–F4, with the sucrose-rich formulation, F2, reaching its limits at the highest storage temperature, i.e., 40 °C. In comparison with sucrose, trehalose exhibits higher glass transition temperatures (T_g_) [53]. In the present study, trehalose-based formulation F3, and even trehalose-rich formulation F4, showed good overall mAb stability and appeared to be a promising approach for the fast and efficient drying of proteins with MFD. The high T_g_ value is also an attribute making cyclodextrins a valuable alternative, with HP-β-CD already being approved in parenteral products [48]. Although the mAb was preserved slightly better in “disaccharide-only” formulations, the authors conclude that the use of an HP-β-CD/sucrose mixture in the described MFD setup is technically possible. Further investigations to find the best excipient ratios are beyond the scope of this study. With regard to the aggressive drying conditions, phosphate was chosen as an arginine counter ion for F6, as it exhibits higher glass transition temperatures compared with others [54]. However, Stärtzel et al. found an increased propensity for the aggregation of an IgG1 mAb in sucrose/arginine phosphate mixtures [55]. After MFD and subsequent storage, we likewise observed protein aggregation and less chemical stability in the protein in F6 compared with other formulations in this study. Consequently, the investigated formulation F6 needs to be optimized further to stabilize the mAb used in this study.

### 3.5. Comparison of the Protein Storage Stability following MFD and CFD

**Figure 4:**
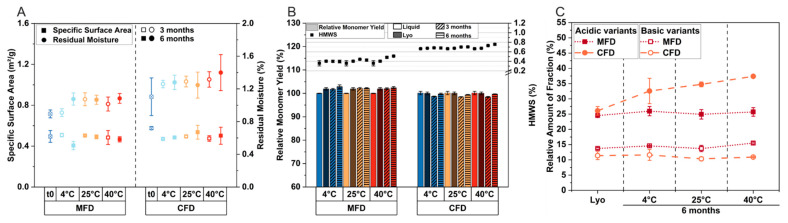
Direct comparison of MFD and CFD: solid-state properties and protein stability for F1 after lyophilization and storage at the respective temperatures over the course of 6 months. In (**A**), the specific surface area (rectangles) and residual moisture (circles) are shown. Relative monomer yield and relative number of high-molecular-weight species (HMWS) are depicted in (**B**). In (**C**), the relative number of acidic and basic variants is shown. All values are means (*n* = 3) ± standard deviation.

To investigate the impact of microwave radiation on degradation, we lyophilized formulation F1 conventionally. The formulation was chosen because it represents a generic composition of a low-concentrated mAb formulation. After freeze-drying, we observed slightly lower residual moisture contents after MFD (0.89% ± 0.05%) compared with CFD (1.10% ± 0.23%), as shown in Figure 4A. Only a slight increase in rM was observed over the course of 6 months at 4 °C, 25 °C, and 40 °C (Figure 4A, circles). Although the drying time was reduced by approximately half for MFD, the specific surface area was found to be comparable for the two technologies after lyophilization and subsequent storage (Figure 4A, rectangles), indicating the comparable pore structure of the cakes.

We then compared protein stability following MFD and CFD. The mAb was well preserved, irrespective of the drying procedure. The relative monomer yield remained constant at all storage temperatures (Figure 4B, bars). With an increasing storage temperature, the formation of HMWS increased slightly for MFD (0.36% ± 0.06% after lyophilization and 0.51% ± 0.01% after 6 months at 40 °C), as well as for CFD (0.67% ± 0.01% after lyophilization and 0.76% ± 0.02% after 6 months at 40 °C); see Figure 4B (symbols).

The quantity of both acidic and basic species was similar following MFD and CFD, respectively (Figure 4C). After MFD, the relative number of acidic species was 24.5% ± 0.6% and 13.7% ± 0.3% for basic species. Following CFD, 26.1% ± 1.4% acidic and 11.4% ± 1.3% basic species were found. After storage for 6 months at 40 °C, the relative number of basic species changed slightly for MFD (15.5% ± 0.3%) and did not vary significantly for CFD (10.9% ± 0.1%). The formation of acidic variants during storage at 40 °C was not observed for the MFD sample population (25.7% ± 1.4% after 6 months), while an increase was observed for conventionally dried samples (37.4% ± 0.5% after 6 months at 40 °C). We assume that the overall slightly higher number of HMWS and increased number of acidic variants following CFD compared with MFD is attributable to slightly higher residual moisture levels in CFD samples [56,57,58]. Therefore, we conclude comparable mAb storage stability, which is in good accordance with previous work [26,27].

## 4. Conclusions

In this study, we explored the application of a novel microwave-assisted freeze-drying setup for the lyophilization of biopharmaceutical formulations. Our work is valuable and relevant, as up to now, a machine setup that is in line with GMP requirements has been missing. Besides drastically reducing drying times, we found that the edge vial effect was inversed. Consequently, energy input is mainly driven by microwave radiation, and the technology has the potential to offset conventionally observed disparities in heat transfer. Moreover, we propose simultaneous primary and secondary drying in MFD, which allows for rather aggressive but still robust drying conditions due to the increase in the glass transition temperature as drying progresses. We studied various representative antibody formulations and showed their applicability in the new MFD setup. The charged amino acid system showed inferior capability in stabilizing the antibody, and it needs to be investigated further. Similar stability profiles were found with MFD vs. CFD for a generic antibody formulation over the course of 6 months, despite drastically shortened drying times for MFD. To underline the operationality of the setup, a representative mAb used worldwide was chosen for the study. By virtue of its unique technical setup, utilizing a GMP lyophilizer with small, flat, and even microwave modules, microwave radiation can be added to the process flexibly and on demand. We believe that the presented setup and data offer a significant advance in the time- and cost-saving manufacturing of essential medicines and represent a crucial step toward the application of the MFD technology to the pharmaceutical industries.

## Figures and Tables

**Table 1:** Investigated formulations with the corresponding drying times as well as the relative monomer yield (RMY) and relative amount of high-molecular-weight species (HMWS) after the storage of the respective mAb formulations.

Formulation Number	Protein Conc. (g/L)	Sucrose (%)	Trehalose (%)	HP-β-CD (%)	Arginine Phosphate (%)	PS 20 (%)	Drying Time (h)	RMY after 6 Months at 40 °C (%)	HMWS after 6 Months at 4 °C (%)	HMWS after 6 Months at 40 °C (%)
F1	10	8.0				0.04	28.5	102.4 ± 0.5	0.40 ± 0.03	0.51 ± 0.01
F2	10	16.0				0.04	26.4	75.5 ± 0.2	0.37 ± 0.01	21.07 ± 0.08
F3	10		8.0			0.04	29.1	102.9 ± 0 8	0.52 ± 0.00	0.73 ± 0.01
F4	10		16.0			0.04	26.9	104.7 ± 0.2	0.58 ± 0.01	0.64 ± 0.01
F5	10	2.4		5.6		0.04	29.9	103.2 ± 0.3	0.70 ± 0.07	1.09 ± 0.02
F6	10				8.0	0.04	31.5	101.1 ± 0.9	1.56 ± 0.11	3.34 ± 0.06

The values are means (*n* = 3) ± standard deviation. HP-β-CD, (2-Hydroxypropyl)-β-cyclodextrin; PS 20, polysorbate 20.

**Table 2:** Relative number of acidic and basic variants after MFD and storage at the respective temperatures over the course of 6 months for all formulations.

Formulation Number	Acidic Variants (%)				Basic Variants (%)			
	0 m	6 m			0 m	6 m		
		4 °C	25 °C	40 °C		4 °C	25 °C	40 °C
F1	24.5 ± 0.6	26.9 ± 1.5	24.9 ± 1.6	25.7 ± 1.4	13.7 ± 0.3	14.6 ± 0.2	13.7 ± 1.0	15.5 ± 0.3
F2	25.8 ± 0.1	27.1 ± 1.4	26.1 ± 2.6	30.5 ± 1.6	13.1 ± 0.3	13.0 ± 0.6	13.4 ± 0.5	20.9 ± 0.7
F3	26.0 ± 0.1	25.5 ± 1.0	26.6 ± 0.6	28.3 ± 1.0	14.5 ± 0.3	13.2 ± 0.2	15.1 ± 0.5	20.5 ± 0.6
F4	24.3 ± 0.6	26.6 ± 2.2	26.3 ± 2.9	28.1 ± 2.4	11.0 ± 0.6	13.5 ± 0.4	14.2 ± 0.4	18.2 ± 1.4
F5	27.8 ± 0.1	29.8 ± 0.3	31.0 ± 0.5	32.9 ± 0.8	11.6 ± 0.6	13.5 ± 1.2	16.3 ± 0.5	25.7 ± 1.8
F6	27.2 ± 1.0	29.0 ± 1.1	31.2 ± 0.4	43.7 ± 0.2	11.8 ± 1.9	12.1 ± 0.4	16.4 ± 0.5	21.4 ± 0.3

The values are means (*n* = 3) ± standard deviation. m, month.

## Data Availability

Data are contained within the article.

## Supplementary Materials

The following supporting information can be downloaded at https://www.mdpi.com/article/10.3390/pharmaceutics15051342/s1: Figure S1: Headspace moisture data of MFD samples determined with frequency-modulated spectroscopy. The formulation contained 8% (m/V) sucrose and 0.04% polysorbate 20 in 10 mM histidine buffer. Values are means (*n* = 27 for edge vials, *n* = 32 for center vials) ± standard deviation. Asterisk (*) indicates statistical significance, *p* < 0.05.; Table S1: Storage stability of the mAb following MFD. Relative monomer yield (RMY) and the relative number of high-molecular-weight species (HMWS) after storage at 4 °C for the respective mAb formulations. The values are means (*n* = 3) ± standard deviation. Table S2: Storage stability of the mAb following MFD. Relative monomer yield (RMY) and the relative number of high-molecular-weight species (HMWS) after storage at 25 °C for the respective mAb formulations. The values are means (*n* = 3) ± standard deviation. Table S3: Storage stability of the mAb following MFD. Relative monomer yield (RMY) and the relative number of high-molecular-weight species (HMWS) after storage at 40 °C for the respective mAb formulations. The values are means (*n* = 3) ± standard deviation. Figure S2: Scanning electron microscopy images from the tops and bottoms of the cakes of F5 and F6 at 175-fold magnification. Figure S3: Characterization of the cake structure with micro-computed tomography (µCT). (A) Average pore size is indicated by the structure separation. (B) Representative µCT pictures of F1 and F3. The rectangular box indicates the analyzed volume of interest (VOI), and the color scale represents the respective pore sizes (dark blue to green: 6 µm to 170 µm ± 6 µm, respectively). Figure S4: Representative X-ray powder diffractograms of the investigated formulations after MFD. Adhesive tape was used to seal the sample holders immediately after sample mounting in order to protect the moisture-sensitive powders from the surrounding air. AU, arbitrary units. Figure S5: Subvisible particle counts for the investigated formulations after MFD and storage at 4 °C ((A), (B), (C)) and 25 °C ((D), (E), (F)) as well as 40 °C ((G), (H), (I)). The values are means (n = 3 and technical duplicates per vial) ± standard deviation.
