# Supplementary material for: Accelerated Production of Biopharmaceuticals via Microwave-Assisted Freeze-Drying (MFD)
Source: Pharmaceutics. 2023 Apr 27;15(5):1342. doi: 10.3390/pharmaceutics15051342 (PMC10223035; doi:10.3390/pharmaceutics15051342)
Supplement: Supplementary file 1 [file pharmaceutics-15-01342-s001.zip › pharmaceutics-2301797-supplementary.pdf]

pharmaceutics

Supplementary Materials: Accelerated Production of Biopharmaceuticals via Microwave–Assisted Freeze–Drying (MFD)

Nicole Härdter, Raimund Geidobler, Ingo Presser and Gerhard Winter

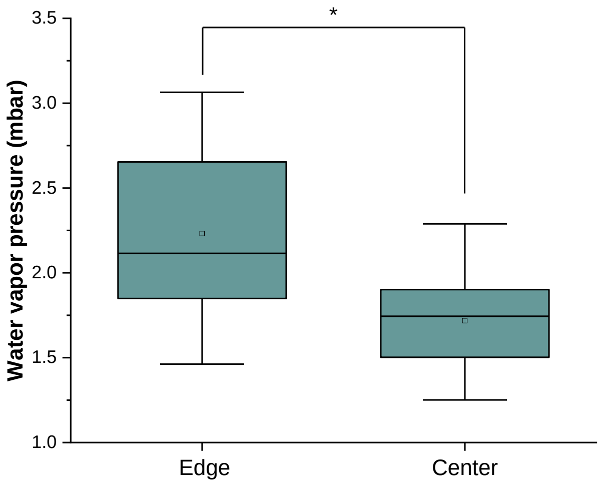

Figure S1. Headspace moisture data of MFD samples determined with frequency modulated spectroscopy. The formulation contained 8% (m/V) sucrose, 0.04% polysorbate 20 in 10 mM histidine buffer. Values are means (n = 27 for edge vials, n = 32 for center vials) ± standard deviation. Asterisk (*) indicates statistical significance, $p < 0.05$.

Table S1. Storage stability of the mAb following MFD. Relative monomer yield (RMY) and relative number of high molecular weight species (HMWS) after storage at 4°C of the respective mAb formulations.

Formulation number	0 m		3 m		6 m	
	RMY, %	HMWS, %	RMY, %	HMWS, %	RMY, %	HMWS, %
F1	101.9 ± 0.5	0.40 ± 0.02	101.8 ± 0.4	0.40 ± 0.03	102.9 ± 0.8	0.40 ± 0.03
F2	105.5 ± 0.1	0.38 ± 0.00	105.6 ± 0.3	0.38 ± 0.00	105.8 ± 0.3	0.37 ± 0.01
F3	102.6 ± 0.3	0.46 ± 0.02	102.2 ± 0.1	0.45 ± 0.06	102.6 ± 0.1	0.52 ± 0.00
F4	104.7 ± 0.6	0.57 ± 0.01	104.9 ± 0.1	0.63 ± 0.00	104.6 ± 0.2	0.58 ± 0.01
F5	103.7 ± 0.6	0.51 ± 0.03	102.0 ± 0.2	0.62 ± 0.02	103.0 ± 0.2	0.70 ± 0.07
F6	99.3 ± 0.6	1.54 ± 0.11	101.2 ± 0.8	1.52 ± 0.17	102.4 ± 1.9	1.56 ± 0.11

The values are means (n = 3) ± standard deviation.

Table S2. Storage stability of the mAb following MFD. Relative monomer yield (RMY) and relative number of high molecular weight species (HMWS) after storage at 25°C of the respective mAb formulations.

Formulation number	0 m		3 m		6 m	
	RMY, %	HMWS, %	RMY, %	HMWS, %	RMY, %	HMWS, %
F1	101.9 ± 0.5	0.40 ± 0.02	102.2 ± 0.4	0.44 ± 0.01	102.2 ± 0.3	0.43 ± 0.02
F2	105.5 ± 0.1	0.38 ± 0.00	105.5 ± 0.7	0.39 ± 0.01	105.9 ± 0.8	0.50 ± 0.21
F3	102.6 ± 0.3	0.46 ± 0.02	102.3 ± 0.3	0.54 ± 0.01	102.9 ± 0.2	0.62 ± 0.01
F4	104.7 ± 0.6	0.57 ± 0.01	104.9 ± 0.2	0.63 ± 0.01	104.7 ± 0.1	0.61 ± 0.01
F5	103.7 ± 0.6	0.51 ± 0.03	102.6 ± 1.4	0.73 ± 0.01	103.1 ± 0.2	0.88 ± 0.02
F6	99.3 ± 0.6	1.54 ± 0.11	101.0 ± 1.4	2.09 ± 0.05	101.5 ± 0.6	2.21 ± 0.08

The values are means (n = 3) ± standard deviation.

Table S3. Storage stability of the mAb following MFD. Relative monomer yield (RMY) and relative number of high molecular weight species (HMWS) after storage at 40°C of the respective mAb formulations.

Formulation number	0 m		3 m		6 m	
	RMY, %	HMWS, %	RMY, %	HMWS, %	RMY, %	HMWS, %
F1	101.9 ± 0.5	0.40 ± 0.02	102.0 ± 0.4	0.48 ± 0.02	102.4 ± 0.5	0.51 ± 0.01
F2	105.5 ± 0.1	0.38 ± 0.00	82.1 ± 1.0	23.27 ± 1.63	75.5 ± 0.2	21.07 ± 0.08
F3	102.6 ± 0.3	0.46 ± 0.02	102.8 ± 0.2	0.62 ± 0.01	102.9 ± 0.8	0.73 ± 0.01
F4	104.7 ± 0.6	0.57 ± 0.01	104.7 ± 0.1	0.67 ± 0.02	104.7 ± 0.2	0.64 ± 0.01
F5	103.7 ± 0.6	0.51 ± 0.03	102.3 ± 0.3	0.93 ± 0.01	103.3 ± 0.3	1.09 ± 0.02
F6	99.3 ± 0.6	1.54 ± 0.11	99.9 ± 0.2	2.93 ± 0.10	101.1 ± 0.9	3.34 ± 0.06

The values are means (n = 3) ± standard deviation.

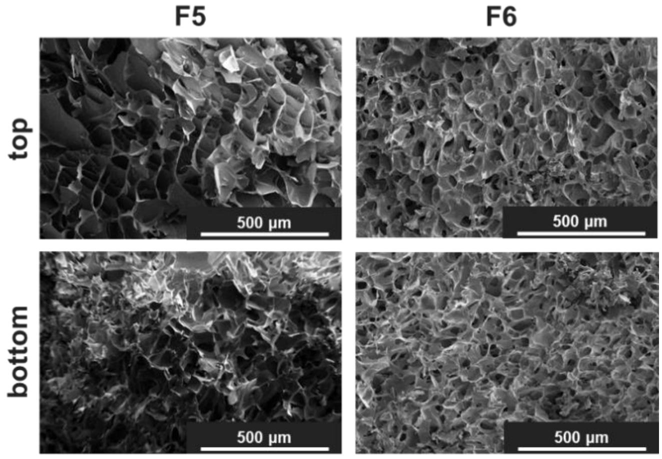

Figure S2. Scanning electron microscopy images from top and bottom of the cakes of F5 and F6 at 175-fold magnification.

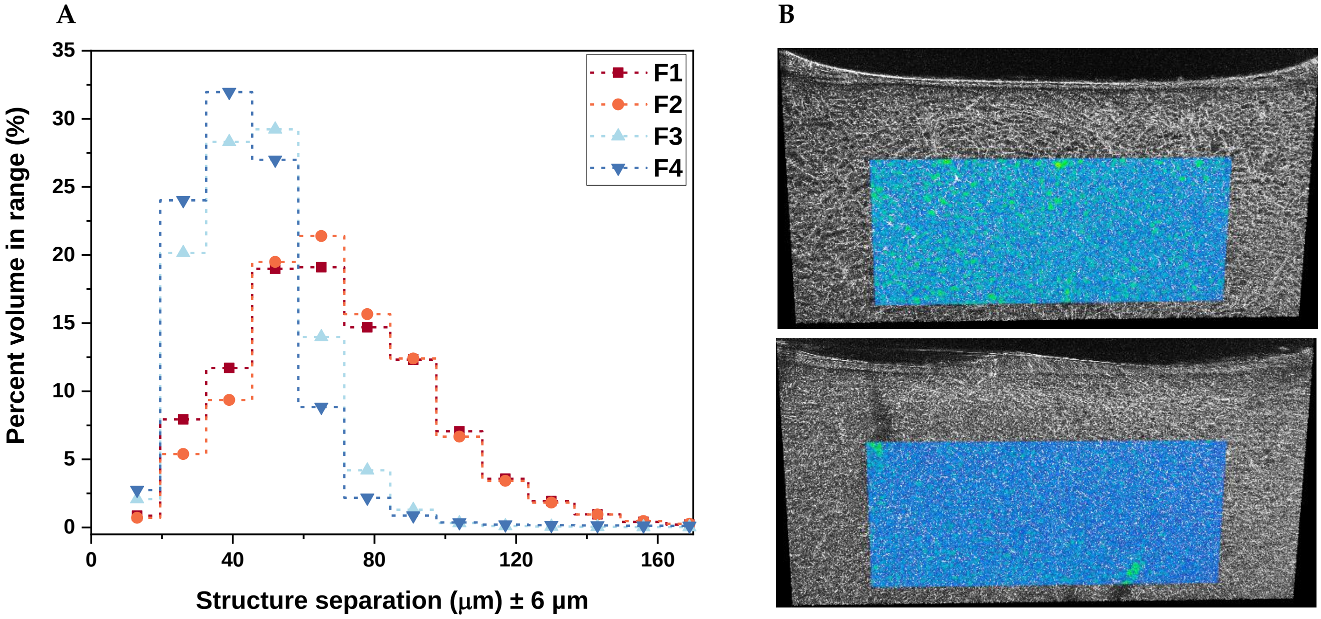

Figure S3. Characterization of the cake structure with micro-computed tomography (µCT). (A) Average pore size is indicated by the structure separation. (B) Representative µCT pictures of F1 and F3. The rectangular box indicates the analyzed volume of interest (VOI) and the color scale represents the respective pore sizes (dark blue to green, 6 µm to 170 µm ± 6 µm, respectively).

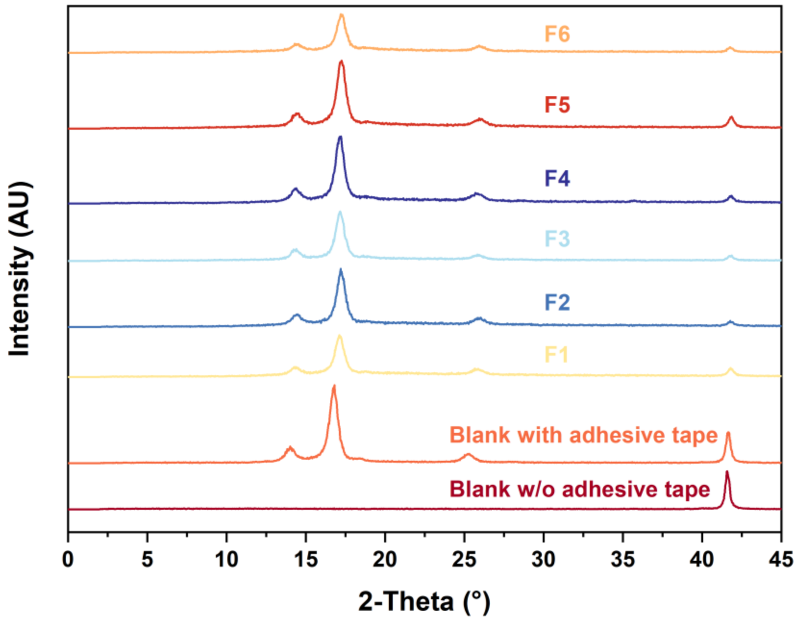

Figure S4. Representative X-ray powder diffractograms of the investigated formulations after MFD. Adhesive tape was used to seal the sample holders immediately after sample mounting in order to protect the moisture sensitive powders from surrounding air. AU, arbitrary units.

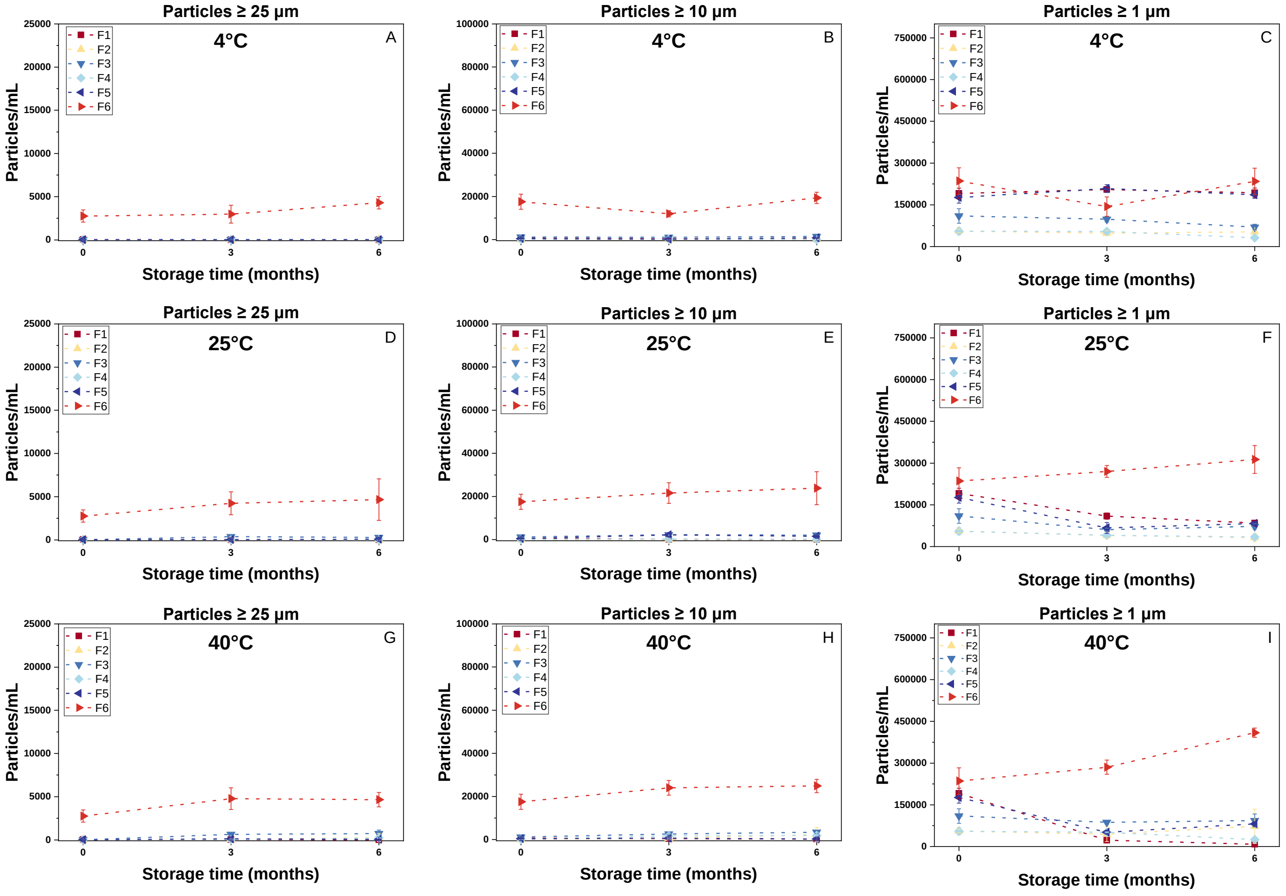

Figure S5. Subvisible particle counts for the investigated formulations after MFD and storage at 4 °C (A), (B), (C) and 25 °C (D), (E), (F) as well as 40 °C (G), (H), (I). The values are means (n = 3 and technical duplicates per vial) ± standard deviation.
